# Panax Notoginseng Saponins Suppress Type 2 Porcine Reproductive and Respiratory Syndrome Virus Replication *in vitro* and Enhance the Immune Effect of the Live Vaccine JXA1-R in Piglets

**DOI:** 10.3389/fvets.2022.886058

**Published:** 2022-05-10

**Authors:** Heyou Yi, Zhiqing Yu, Qiumei Wang, Yankuo Sun, Jie Peng, Yu Cai, Jun Ma, Yongjie Chen, Chenxiao Qin, Mengkai Cai, Chihai Ji, Guihong Zhang, Heng Wang

**Affiliations:** ^1^Key Laboratory of Zoonosis Prevention and Control of Guangdong Province, Guangdong Laboratory for Lingnan Modern Agriculture, Guangdong Provincial Key Laboratory of Prevention and Control for Severe Clinical Animal Diseases, College of Veterinary Medicine, South China Agricultural University, Guangzhou, China; ^2^National Engineering Research Center for Breeding Swine Industry, South China Agricultural University, Guangzhou, China; ^3^Key Laboratory of Veterinary Bioproduction and Chemical Medicine of the Ministry of Agriculture, Engineering and Technology Research Center for Beijing Veterinary Peptide Vaccine Design and Preparation, Zhongmu Institutes of China Animal Husbandry Industry Co., Ltd., Beijing, China; ^4^Guangdong Meizhou Vocational and Technical College, Meizhou, China

**Keywords:** PRRSV, PNS, replication, modified live virus vaccine, neutralizing antibodies

## Abstract

Porcine reproductive and respiratory syndrome virus (PRRSV) suppresses the innate immune response in the host, reducing and delaying neutralizing antibody production against PRRSV infection and promoting viral infection. Here, we aimed to assess the potential of Panax notoginseng saponins (PNS) for improving the immune response exerted upon PRRSV-2-modified live virus (MLV) vaccine administration. Thirty piglets were randomly divided into six groups. Group 1 piglets were injected with medium 0 days post vaccination (dpv). Group 2 piglets were fed PNS 0–28 dpv. Group 3 and group 4 piglets were administered the JXA1-R vaccine 0 dpv. Group 4 piglets were also fed PNS 0–28 dpv. Group 1–4 piglets were challenged intranasally with the PRRSV JXA1 strain 28 dpv. Group 5 piglets were fed with PNS without challenge. Group 6 piglets served as controls. During the experiment, the samples were collected regularly for 49 days. Compared with group 1 piglets, group 3 piglets showed significantly reduced viremia and clinical scores, and significantly increased average daily gain (ADWG). Compared with group 3 piglets, group 4 piglets showed significantly improved neutralizing antibody titers, IFN-α and IFN-β mRNA expression, and significantly decreased viremia and viral load in the lungs and lymph nodes, but did not demonstrate any further improvement in PRRSV-specific antibody titer, rectal temperature, ADWG, or clinical scores. PNS upregulates neutralizing antibodies against PRRSV-2 and enhances the expression of IFN-α and IFN-β, which may reduce PRRSV viremia upon PRRSV-2 MLV vaccine administration. PNS may serve as an effective immunomodulator for boosting the immune defense against PRRSV.

## Introduction

Porcine reproductive and respiratory syndrome virus (PRRSV) was first discovered in the United States in 1980s. It has been threatening the development of the pig industry in recent decades, resulting in huge economic losses. PRRSV can be divided into genotype 1 represented by European strains and genotype 2 represented by North American strains, which show considerable differences and high variations (1, 2). PRRSV has ~15 kilobases (kb) long genome and contains at least ten open-reading frames, encoding at least fourteen non-structural and seven structural proteins (3).

Currently, vaccination is the main feasible method for controlling PRRSV infection, while commercial vaccines, including inactive vaccines and modified live vaccines (MLVs), fail to provide complete protection (4, 5). At present, the available commercial PRRSV MLV vaccine triggers a delayed and low-level immune response following immunization (6). Neutralizing antibodies play a key role in the fight against PRRSV infection. However, neutralizing antibodies against PRRSV infection are not high in numbers and appear ~3 weeks after vaccination (7). Specific IFN-γ production by T cells, which is a measure of T cell cytotoxicity, appears ~2–4 weeks after vaccination (8). Current PRRSV MLVs do not provide sufficient immune protection (9). There is an urgent need to develop new strategies to control this infectious disease, especially to improve the immune effect of the vaccine against PRRSV.

A variety of natural ingredients found in Chinese herbal medicines possess anti-viral activities *in vitro*, including ginsenoside Rg1 and platycodin D (10, 11), while the compatibility of some natural ingredients, including quill A and quercetin, with vaccines can effectively improve the immune effect by upregulating the expression of interferons (12, 13). Albizia julibrissin saponins (AJSAF) can enhance the numbers of PRRSV N-protein-specific antibodies (14). Because the PRRSV MLV vaccine causes delayed and weak immune response after vaccination, we wondered whether Chinese herbal medicine could adjust the situation.

Panax notoginseng is a traditional Chinese medicinal herb used in China. Panax notoginseng saponins (PNS) are chemical mixtures containing different types of adamantane saponins extracted from Panax notoginseng. PNS are the main effective components of Panax notoginseng. PNS can be biotransformed into bioactive metabolites, such as notoginsenoside R2, ginsenoside Rg1, ginsenoside Rh1, ginsenoside F1, and protopanaxatriol (PPT), by the gut microbiota (15). Previous studies have shown that PNS has anti-inflammatory, anti-viral, and immunomodulatory activities. PNS regulates the proliferation and differentiation of Th17 cells by reducing the generation of inflammatory factors (16). Reportedly, notoginsenoside R1 significantly inhibits the expression of p-IκBα and NF-κB P65 (17). Notoginsenoside ST-4 can block the penetration of the herpes simplex virus, thereby inhibiting virus replication (18). PNS can promote the proliferation of hematopoietic progenitor cells and modulate T cell immune functions (19). PNS can inhibit the oxidative stress of immune cells when infected with PCV2 (20). At present, research on the anti-viral activity of PNS is still in its infancy, and its immunomodulatory effects have not been evaluated. Understanding its effects may lead to the exploitation of how PNS help to combat PRRSV in the pig industry.

The objective of this study was to evaluate the inhibitory activity of PNS on PRRSV *in vitro* and *in vivo*, as well as its effectiveness as an immune adjuvant on the immune response of the PRRS vaccine strain JXA1-R.

## Materials and Methods

### Viral Strains and Cells

The commercial attenuated live vaccine JXA1-R strain was purchased from Guangdong Winsun Bio-Pharmaceutical Co., Ltd. PRRSV GM2-like strain (lineage3) and NADC30-like strain (lineage1) were isolated from sows with reproductive problems and preserved in our lab. The PRRSV JXA1 strain (GenBank accession no. EF112445.1) was presented by Professor Tian. Marc-145 cells used in this study were obtained from the stocks in our laboratory.

### Cytotoxicity Assay

Marc-145 cells were cultured in Dulbecco's modified Eagle medium (DMEM) containing 2, 4, 8, 16, 32, 64, 128, 256, 512, 1,024, 2,048, 4,096, and 8,192 μg/mL PNS (Chengdu Bailunsi Biotechnology Co., Ltd.) for 48 h, and cell viability was analyzed with CCK8 assay (Beyotime Biotechnology, China).

### Quantitative Real-Time PCR

Total RNA was extracted using a total RNA rapid extraction kit (Fastagen, Shanghai, China). RNA was converted into cDNA by MLV (Takara, Japan). qRT-PCR experiments were performed using TB Green® Premix Ex Taq^TM^ II or Premix Ex Taq^TM^ (Probe qPCR, Takara, Japan) in a CFX96 Real-Time System (Bio-Rad, USA). The absolute mRNA expression levels were calculated using standard curves. The relative mRNA expression levels were calculated using the 2^−ΔΔCT^ method. All of primers for PCR amplification are listed in Table 1.

**Table 1:** Real-time PCR primer sequences.

**Name**	**Primer sequence (5^**′**^-3^**′**^)**
Nsp9-F	CCTGCAATTGTCCGCTGGTTTG
Nsp9-R	GACGACAGGCCACCTCTCTTAG
Nsp9 Probe	ACTGCTGCCACGACTTACTGGTCACGCAGT
GAPDH-F	CCTTCCGTGTCCCTACTGCCAAC
GAPDH-R	GACGCCTGCTTCACCACCTTCT
IFNα-F	GGCTCTGGTGCATGAGATGC
IFNα-R	CAGCCAGGATGGAGTCCTCC
IFNβ-F	AGTTGCCTGGGACTCCTCAA
IFNβ-R	CCTCAGGGACCTCAAAGTTCAT
TNFα-F	CCAATGGCAGAGTGGGTATG
TNFα-R	TGAAGAGGACCTGGGAGTAG

### Anti-viral Activity Assay

To analyze the effect of PNS on PRRSV infection, Marc-145 cells grown to a single layer in 6-well plates were inoculated with MOI of 0.05 TCID_50_ per cell of PRRSV at 37°C for 1 h. Supernatants were discarded and fresh DMEM containing various concentrations of PNS was added. After 48 h of treatment, supernatant and cells were collected for virus titer determination and western blotting analysis. The concentration required for 50% of the maximum effect (EC_50_) to be observed was determined as previously described (11), and was analyzed using GraphPad Prism 7.0.

### Western Blotting Analysis

Cells were lysed in NP40 lysis buffer (Beyotime Biotechnology, China) with 1% phenylmethylsulfonyl fluoride (Beyotime Biotechnology, China). The samples were separated via SDS-PAGE (15%) and transferred to a nitrocellulose membrane (Millipore, USA). The nitrocellulose membrane was blocked with 5% milk (BD, USA) and then incubated with mouse anti-N-protein monoclonal antibodies (SDOW17, Korea). Goat anti-mouse IgG (LI-COR Biosciences, USA) was used as the secondary antibody for 1 h at room temperature. The Odyssey system (LI-COR Biosciences, USA) was used to analyze the membranes.

### Multistep Growth Curve

Briefly, Marc-145 cells, which were grown to a single layer in 6-well plates, were infected with PRRSV (MOI of 0.05 TCID_50_ per cell) for 1 h. Then, the supernatant of each well was removed and replaced with fresh DMEM containing 32 or 64 μg/mL of PNS. The supernatants were collected again 4, 12, 24, 36, 48, 60, 72, 84, 96, 108, and 120 h post-infection (hpi) to determine viral titers, which were defined as the median tissue culture infectious dose (TCID_50_).

### Effect of PNS Treatment on the PRRSV Life Cycle

Marc-145 cells grown to a single layer in 6-well plates were incubated with PRRSV (MOI of 0.05 TCID_50_ per cell). PRRSV life cycle, including attachment, internalization, replication, and release, was analyzed at indicated time points after infection, and the effect of pre-treatment with various concentrations of PNS on PRRSV infection was analyzed as previously described (11).

### Animal Experiments

Thirty-four-week-old piglets, which were free of PRRSV and antibodies against PRRSV were randomly divided into six groups. Group 1 piglets were orally treated with 5 mL of DMEM 0–28 dpv. Group 2 piglets were orally treated with PNS [10 mg/kg bodyweight (bw)] dissolved in DMEM (5 mL) 0–28 dpv. Group 3 piglets were intramuscularly injected with JXA1-R attenuated live vaccine (TCID_50_ of 10^8^) 0 days post vaccination (dpv) and orally treated with 5 mL of DMEM 0–28 dpv. Group 4 piglets were intramuscularly injected with JXA1-R attenuated live vaccine (10^8^ TCID_50_) 0 dpv and orally treated with PNS (10 mg/kg bw) dissolved in DMEM (5 mL) 0–28 dpv. Group 5 piglets were orally treated with PNS (10 mg/kg bw) dissolved in DMEM (5 mL) 0–28 dpv and used as the control. Group 6 piglets were orally treated with 5 mL of DMEM 0–28 dpv and then used as a negative control.

Before the challenge, venous blood was collected from all piglets to isolate peripheral blood mononuclear cells (PBMCs) and serum. The peripheral blood lymphocytes of each piglet were inoculated with PRRSV (MOI of 0.05 TCID_50_ per cell) for 12, 24, 36, and 48 h to detect the mRNA expression of cytokines IFN-α, IFN-β, and TNF-α. The serum of each piglet was used to titer the neutralizing antibody.

Twenty-eight days post vaccination, all piglets in each group, except for groups 5 and 6, were challenged with HP-PRRSV JXA1 (TCID_50_ of 10^5^) and monitored for 21 days after the challenge. The experimental schedule is presented in Table 2. Blood samples from each piglet were obtained by venipuncture for the detection of the PRRSV viral load and anti-PRRSV antibody concentration (Idexx PRRSV ELISA Kit, Idexx, US). Tissue samples from the lungs, thymus, and lymph nodes were collected 49 dpv. Tissue (1 g), nose swab diluted in PBS (100 μL), and serum (100 μL) samples were used to extract total RNA.

**Table 2:** Summary of experiment schedule.

**Gruop**	***N***	**Vaccine**	**PNS**	**Challenge**	**Antibody detection**	**PBMC isolation**	**Viremia**	**Nasal swabs**	**Rectal temperature**	**Body weight**
1	5	–	–	28 dpv	0, 7, 14, 21, 28, 30, 32, 35, 38, 42, 49 dpv	28 dpv	30, 32, 35, 38, 42, 49 dpv	30, 32, 35, 38, 42, 45, 47, 49 dpv	0–49 dpv	0, 3, 6, 9, 12, 15, 18,21,24, 28, 31, 34, 37, 40, 43, 46, 49 dpv
2	5	–	0–28 dpv	28 dpv	0, 7, 14, 21, 28, 30, 32, 35, 38, 42, 49 dpv	28 dpv	30, 32, 35, 38, 42, 49 dpv	30, 32, 35, 38, 42, 45, 47, 49 dpv	0–49 dpv	0, 3, 6, 9, 12, 15, 18,21,24, 28, 31, 34, 37, 40, 43, 46, 49 dpv
3	5	0 dpv	–	28 dpv	0, 7, 14, 21, 28, 30, 32, 35, 38, 42, 49 dpv	28 dpv	30, 32, 35, 38, 42, 49 dpv	30, 32, 35, 38, 42, 45, 47, 49 dpv	0–49 dpv	0, 3, 6, 9, 12, 15, 18,21,24, 28, 31, 34, 37, 40, 43, 46, 49 dpv
4	5	0 dpv	0–28 dpv	28 dpv	0, 7, 14, 21, 28, 30, 32, 35, 38, 42, 49 dpv	28 dpv	30, 32, 35, 38, 42, 49 dpv	30, 32, 35, 38, 42, 45, 47, 49 dpv	0–49 dpv	0, 3, 6, 9, 12, 15, 18,21,24, 28, 31, 34, 37, 40, 43, 46, 49 dpv
5	5	–	0–28 dpv	–	0, 7, 14, 21, 28, 30, 32, 35, 38, 42, 49 dpv	28 dpv	30, 32, 35, 38, 42, 49 dpv	30, 32, 35, 38, 42, 45, 47, 49 dpv	0–49 dpv	0, 3, 6, 9, 12, 15, 18,21,24, 28, 31, 34, 37, 40, 43, 46, 49 dpv
6	5	–	–	–	0, 7, 14, 21, 28, 30, 32, 35, 38, 42, 49 dpv	28 dpv	30, 32, 35, 38, 42, 49 dpv	30, 32, 35, 38, 42, 45, 47, 49 dpv	0–49 dpv	0, 3, 6, 9, 12, 15, 18,21,24, 28, 31, 34, 37, 40, 43, 46, 49 dpv

### Clinical Evaluation

After the piglets received the vaccine, the rectal temperature and the clinical signs were recorded every day. Average daily weight gain (ADWG) was recorded every 3 days from 28 to 49 dpv (equivalent to 0 to 21 days post-challenge). The clinical scores were calculated as previously described (21).

### Neutralization Assay

Serum samples of JXA1-R immunized piglets were collected at 21 dpv and were heat-inactivated at 56°C for 40 min before use. Neutralization assays were performed by mixing 50 μL (TCID_50_ of 100) of JXA1, GM2-like, and NADC30-like strains with 50 μL of serially 2-fold diluted serum and incubated at 37°C for 1 h. The titers were expressed as the highest serum dilutions that could cause a 50% reduction in CPE relative to the control.

### Statistical Analysis

All experiments were performed at least thrice. Data collected from triplicate experiments were represented as the mean ± standard deviation (SD). Statistical analyses were performed using one-way analysis of variance (ANOVA) followed by Tukey's *t*-test with GraphPad Prism 7.0. ^*^P < 0.05, ^**^P < 0.01, ^***^P < 0.001, ^****^P < 0.0001, were considered to be statistically significant.

### Ethics Statement

The animal experiments were approved by the Laboratory Animal Committee of South China Agricultural University (SCAU, No. 2019C019). All operations were performed in accordance with the animal ethics guidelines and approved protocols.

## Results

### PNS Treatment Suppresses PRRSV Replication in Marc-145 Cells

**Figure 1:**
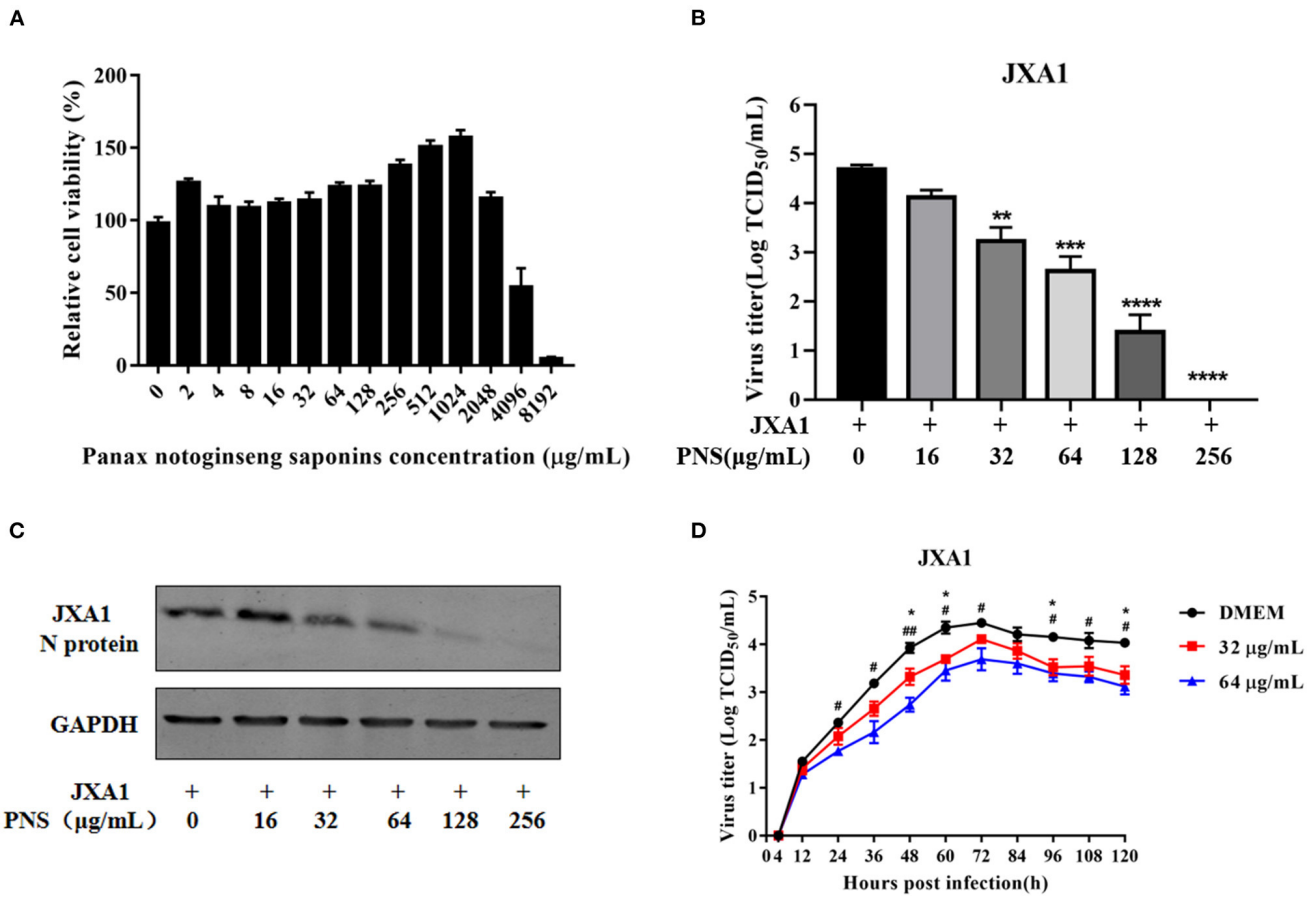
Cytotoxicity and anti-PRRSV activity of PNS in Marc-145 cells. **(A)** Determination of PNS cytotoxicity in Marc-145 cells. Cells were incubated with 0, 2, 4, 8, 16, 32, 64, 128, 256, 512, 1,024, 2,048, 4,096, and 8,192 μg/mL PNS for 48 h before CCK8 assay. **(B,C)** Marc-145 cells were treated with PNS at 0, 16, 32, 64, 128, and 256 μg/mL for 48 h after JXA1 infection (MOI of 0.05 TCID_50_ per cell) for 1 h. The infected cells were cultured in the presence of PNS at different concentrations. The cells and supernatant were harvested at 48 hpi for the TCID_50_ assay and western blotting analysis. Western blotting analysis was performed using PRRSV N-protein antibody, and GAPDH served as loading control. **(D)** Marc-145 cells were incubated with JXA1 strains (MOI of 0.05 TCID_50_ per cell) for 1 h at 37°C in DMEM supplemented with 0, 16, 32, 64, 128, and 256 μg/mL PNS. Supernatants were collected 4, 12, 24, 36, 48, 60, 72, 84, 96, 108, 120 h after inoculation for virus titer determination. Statistical significance is denoted by *p < 0.05, **p < 0.01, ***p < 0.001. #*p* < 0.05, ##*p* < 0.01, ****p < 0.0001.

The cytotoxicity of PNS on Marc-145 cells was determined using CCK8 assay. As shown in Figure 1A, 4.5–53.7% Marc-145 cells treated with 4,096 μg/mL or higher concentrations of PNS were viable, and the highest safe concentration of PNS on Marc-145 cells was 2,048 μg/mL. The viable Marc-145 cells treated with PNS within the range of 4–1,024 μg/mL grew in a dose-dependent manner.

To determine the anti-PRRSV activity of PNS, Marc-145 cells were infected with PRRSV JXA1 (MOI of 0.05 TCID_50_ per cell) for 1 h, followed by incubation with 0, 16, 32, 64, 128, and 256 μg/mL PNS for 48 h. As shown in Figure 1B, the virus titer and the expression of the N-protein indicated that PNS treatment significantly inhibited PRRSV proliferation in a dose-dependent manner, and 256 μg/mL of PNS completely inhibited PRRSV replication.

### PNS Treatment Affects the Replication Step of the PRRSV Life Cycle in Marc-145 Cells

**Figure 2:**
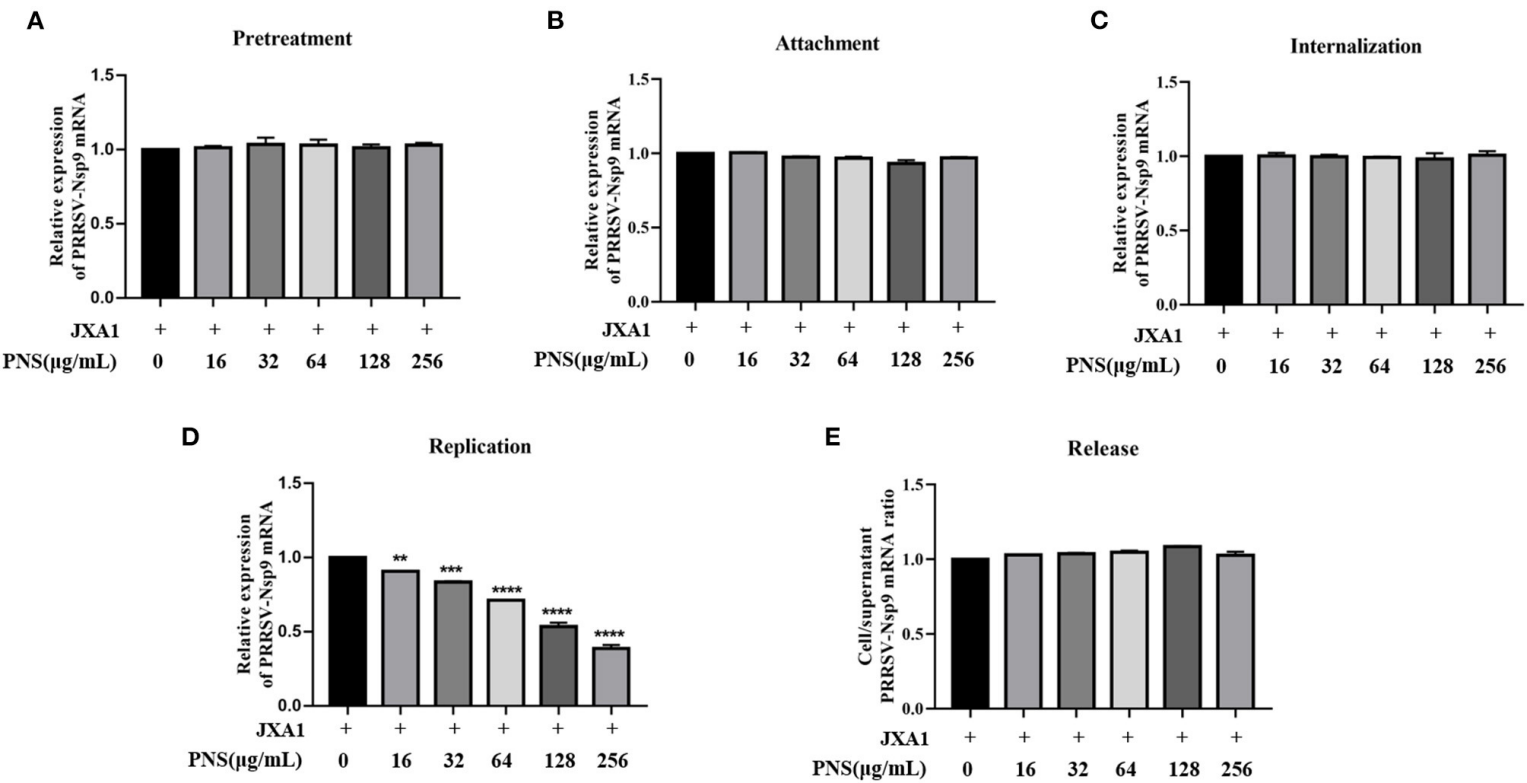
PNS inhibits PRRSV replication. **(A)** Pre-treatment assay. Marc-145 cells were pretreated with 0, 10, 16, 32, 64, 128, 256 μg/mL PNS for 2 h, then, cells were washed with precooled PBS prior to PRRSV JXA1 (MOI of 0.05 TCID_50_ per cell) addition, and then supernatant and cells were collected 48 hpi to detect Nsp9 mRNA expression levels. **(B)** Attachment assay. Marc-145 cells were prechilled at 4°C for 1 h and then the media were replaced by a mixture of various concentrations of PNS and PRRSV (MOI of 0.05 TCID_50_ per cell). After being incubated at 4°C for an additional 2 h, the cells were washed with precooled PBS, and then qRT-PCR was performed. **(C)** Internalization assay. Marc-145 cells were prechilled at 4°C for 1 h and then incubated for another 2 h at 4°C with PRRSV (MOI of 0.05 TCID_50_ per cell). After washing three time with precooled PBS, cells were placed in medium with various concentrations of PNS and the temperature was increased to 37°C for 3 h. qRT-PCR was then performed. **(D)** Replication assay. Marc-145 cells were infected with PRRSV (MOI of 0.05 TCID_50_ per cell) for 6 h and then washed three times with precooled PBS. The cells were cultured in fresh media containing various concentrations of PNS and collected 4 hpi for qRT-PCR. **(E)** Release assay. Marc-145 cells were incubated with PRRSV (MOI of 0.05 TCID_50_ per cell) for 1 h and then were replaced by the fresh medium for 24 h. The cells were cultured in a fresh medium with various concentrations of PNS. The cells and supernatants were harvested 2 h after the medium switch for qRT-PCR. Statistical significance is denoted by **p < 0.01, ***p < 0.001, ****p < 0.0001.

To explore the effect of PNS treatment on PRRSV replication in Marc-145 cells, virus attachment, internalization, replication, and release were analyzed as previously described (11). The viral RNA levels in the supernatant or cells were represented by PRRSV Nsp9 determined using qRT-PCR. The results indicated that pre-treatment with PNS did not affect the susceptibility of Marc-145 cells to PRRSV (Figure 2A). Furthermore, PNS treatment at concentrations of 0–256 μg/mL, did not affect PRRSV attachment (Figure 2B), internalization (Figure 2C), or release (Figure 2E). The replication rate of PRRSV was significantly inhibited upon PNS treatment in a dose-dependent manner (Figure 2D).

### PNS Treatment Suppresses the Viral Reproduction of the JXA1-R, NADC30-Like, and GM2-Like Strains in Marc-145 Cells

**Figure 3:**
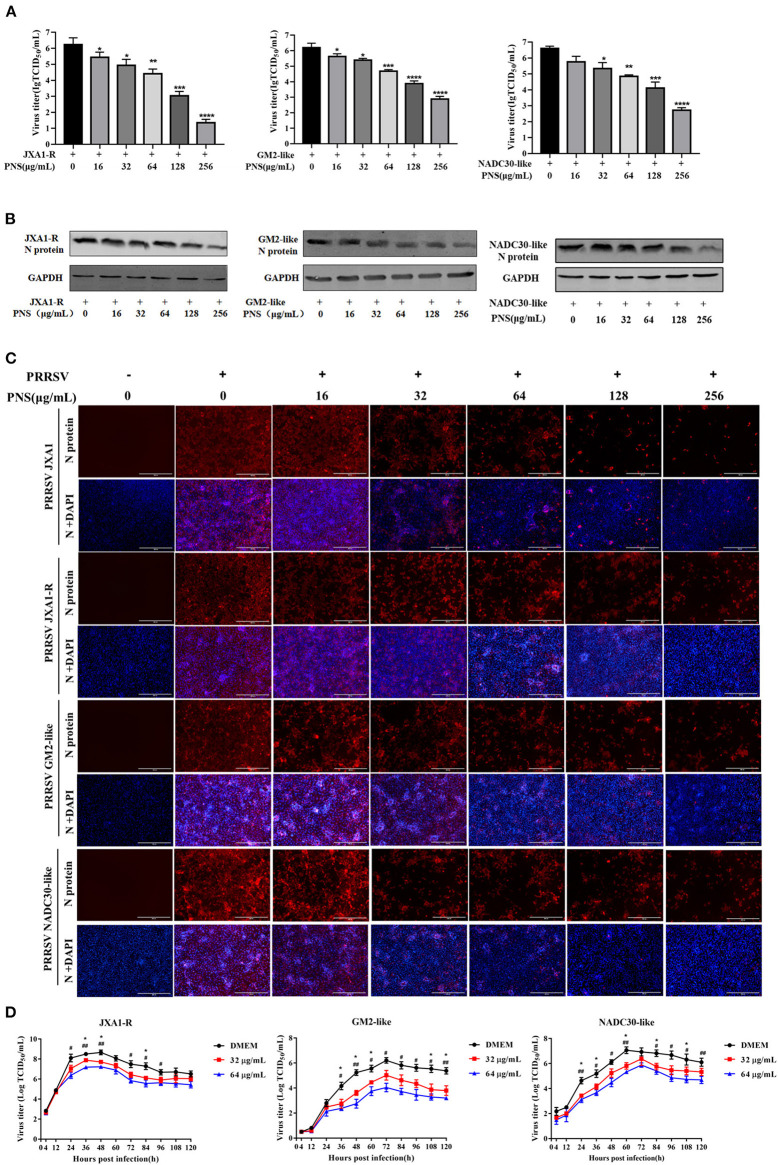
The anti-viral activity of PNS against different lineages of type 2 PRRSV. **(A–C)** Anti-viral activity of PNS against PRRSV strains (JXA1-R, GM2-like, and NADC30-like) was assessed in Marc-145 cells. Marc-145 cells were treated with PNS at 0, 16, 32, 64, 128, and 256 μg/mL for 48 h after PRRSV infection (MOI of 0.05 TCID_50_ per cell) for 1 h. The samples were collected for the TCID_50_ assay **(A)**, western blotting **(B)**, and IFA **(C)**. Western blotting was performed using PRRSV N-protein antibody, and GAPDH served as loading control. Marc-145 cells were immunostained for the PRRSV N-protein antibody with an Alexa Fluor 594-conjugated goat anti-mouse antibody (Red). **(D)** Marc-145 cells were infected with PRRSV (JXA1-R, GM2-like, NADC30-like) (MOI of 0.05 TCID_50_ per cell) for 1 h at 37°C and cultured in DMEM supplemented with indicaited concentrations of PNS. Supernatants were collected 4, 12, 24, 36, 48, 60, 72, 84, 96, 108, 120 hpi for virus titer determination. Statistical significance is denoted by *p < 0.05, **p < 001, ***p < 0.001, ****p < 0.0001. #*p* < 0.05, ##*p* < 0.01.

To determine whether PNS has anti-viral activity against other lineages of PRRSV or the commercial live vaccine, Marc-145 cells were infected with JXA1-R, NADC30-like (lineage 1), and GM2-like strains (lineage 3) individually, followed by PNS gradient treatment. The virus titer, the expression level of the N-protein, and the IFA results revealed that the replication of JXA1-R, NADC30-like, and GM2-like strains in Marc-145 cells decreased upon PNS treatment in a dose-dependent manner, but the inhibitory effect was different (Figures 3A–C). Therefore, the EC_50_ against three PRRSV strains in Marc-145 cells ranged from 65.06 to 113.53 μg/mL, calculated using IFA images (Table 3).

**Table 3:** Inhibitory activity of PNS against PRRSV infection in Marc-145 cells.

	**PRRSV strain**			
	**JXA1**	**JXA1-R**	**GM2-like**	**NADC30-like**
EC_50_(μg/mL)^a^	65.06 ± 13.35	84.37 ± 22.47	113.53 ± 23.21	101.87 ± 19.04

a *The concentration required to protect 50% cells from PRRSV infection was determined by plotting the relative infected-cell percentage from IFA images as a function of compound concentration and calculated with the GraphPad Prism 5.0 software*.

To assess the suppressive effect of PNS on the replication of the three PRRSV strains, the growth curves were determined. The results indicated that the inhibitory effect of PNS on progeny virus was mainly at the plateau phase, and the anti-viral activity was most obvious at 64 μg/mL (Figure 3D).

### PNS Treatment Did Not Affect the Clinical Behavior and Anti-PRRSV Antibody Levels in Vaccinated Piglets

**Figure 4:**
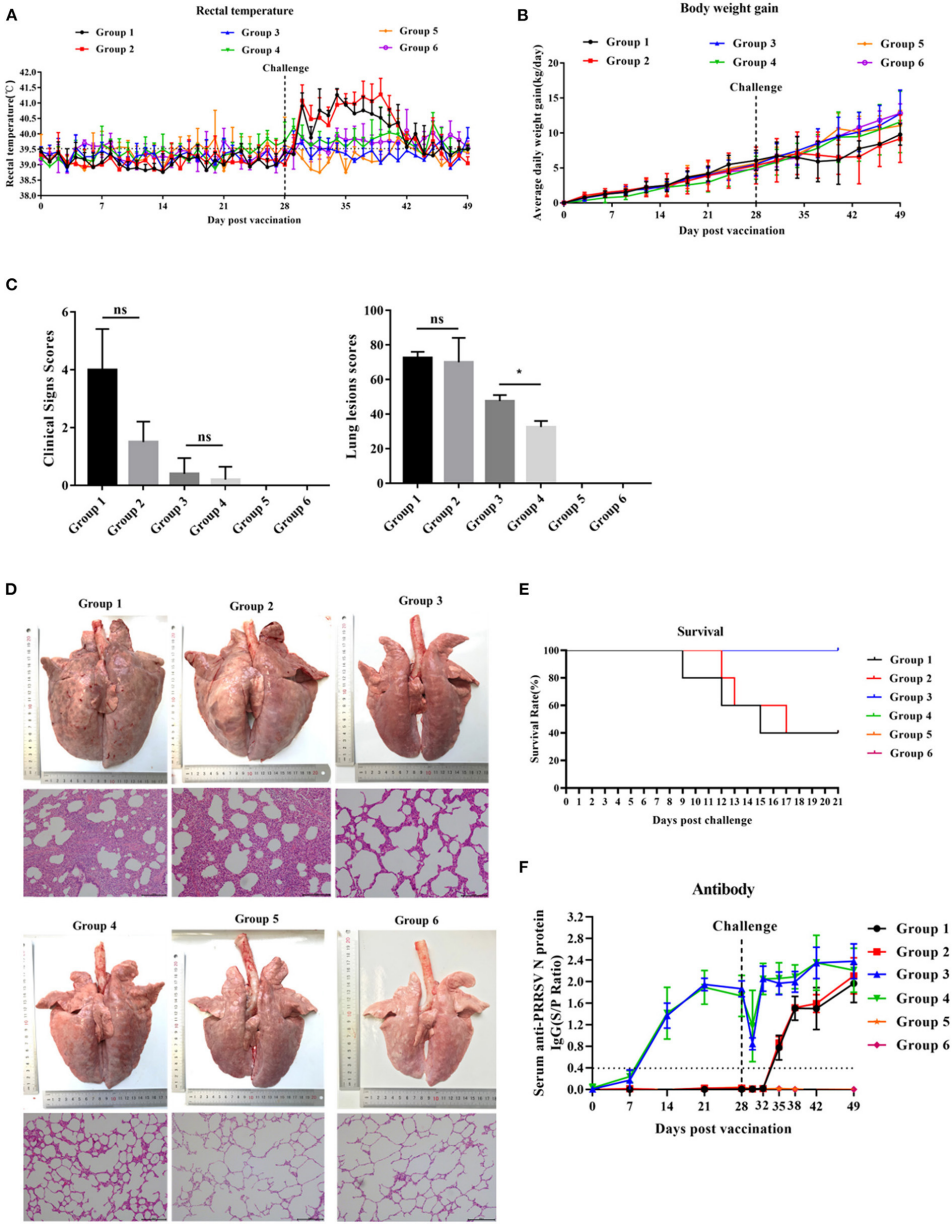
The effect of PNS on vaccine immunity. **(A)** Changes in daily rectal temperature of piglets inoculated with highly virulent PRRSV JXA1. **(B)** The change in the weight of piglets after challenge. **(C)** Changes in the lungs of piglets in different groups 21 dpc. **(D)** The scores of clinical signs and lung lesions of piglets in different groups 21 dpc. **(E)** Survival curves of piglets infected with JXA1 in each group. **(F)** The anti-PRRSV antibody levels in serum. The S/P ratio of ≥ 0.4 was considered antibody positive. Statistical significance is denoted by *p < 0.05, ns means no significant difference.

After HP-PRRSV JXA1 strain challenge, groups 1 and 2 exhibited high rectal temperature (Figure 4A) and showed typical clinical features of HP-PRRS, such as anorexia, cough, dyspnea, and lameness. Group 1 and group 2 piglets had rectal temperatures above 40°C from 2 to 13 days post-challenge (dpc), respectively, with a peak temperature of 41.2°C 6 and 11 dpc. The peak rectal temperature observed in group 2 piglets was 5 days later than that observed in group 1 piglets (Figure 4A). The rectal temperature of group 3 and group 4 piglets was maintained between 39.0 and 40°C, but the rectal temperature of group 4 piglets was slightly higher than that of group 3 piglets. Piglets in groups 5 and 6 behaved normally throughout the experiment. The ADWG of group 1 piglets began to decrease later than that of group 2 piglets (Figure 4B). ADWG in the other groups was not abnormal. Clinical sign scores were not significantly different among the groups 21 dpc (Figure 4C).

As shown in Figure 4D, the lungs of group 1 and 2 piglets displayed severe gross lesions with hemorrhagic consolidation, dense parenchymal striae, and hemorrhage compared to group 1. Three of the five piglets in groups 1 and 2 died between days 9 and 17 post-challenge, but the death time of piglets in group 2 was slightly later than that in group 1 (Figure 4E). Lung lesion scores in group 4 were significantly lower than those in group 3 (Figure 4C). Group 3 piglets showed moderate interstitial pneumonia and thickened alveolar walls, while group 4 piglets exhibited mildly thickened alveolar walls. No lesions were observed in groups 5 and 6 (Figure 4D).

All piglets in groups 3 and 4 developed anti-PRRSV N-protein antibodies 14 dpv, and the antibody concentration peaked 42 dpv (Figure 4F). No significant difference in the S/P ratio between piglets in groups 3 and 4 was detected throughout the experiment. Piglets in groups 1 and 2 developed anti-PRRSV N-protein antibody 35 dpv, and no significant difference in the S/P ratio was detected (Figure 4F). Piglets in groups 5 and 6 remained negative for PRRSV throughout the experiment.

### Vaccinated Piglets Treated With PNS Possessed High Concentrations of PRRSV-Neutralizing Antibodies

**Figure 5:**
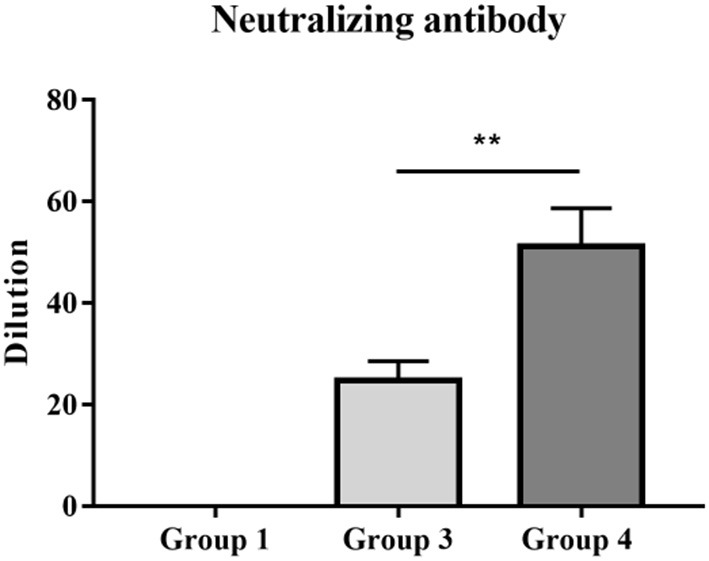
PRRSV titers of vaccinated piglets with anti-sera. The neutralizing activities of serum samples collected 21 dpv were detected against homologous JXA1 strain. Statistical significance is denoted by **p < 0.01.

Twenty-one days after immunization, anti-serum samples were collected from JXA1-R-treated piglets and their neutralization capacity against JXA1, GM2-like, and NADC30-like strains was determined. The virus neutralization titer of group 4 piglets against JXA1 was significantly higher than that of group 3 piglets (Figure 5). However, there was no neutralization against GM2-like or NADC30-like strains in group 3 and 4 piglets (data not shown). In summary, PNS provided neutralization protection against homologous strains.

### PNS Treatment Significantly Reduces HP-PRRSV Viremia and Tissue Viral Load of Vaccinated Piglets

**Figure 6:**
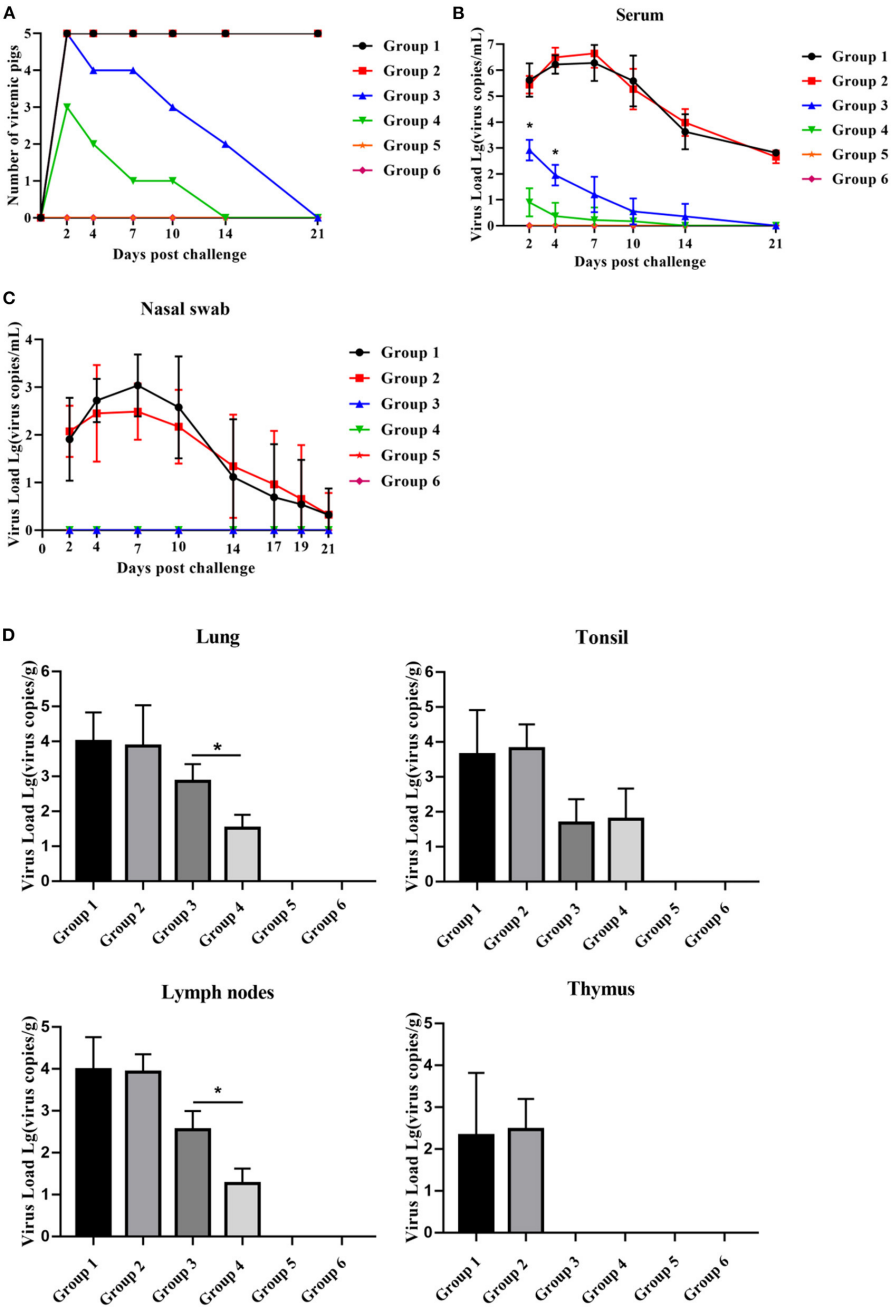
PNS treatment significantly reduced HP-PRRSV viremia and tissue viral load. **(A)** The number of viremic pigs. **(B)** The level of PRRSV mRNA in serum, detected using qRT-PCR. The expression levels of PRRSV mRNA in nasal swabs **(C)** lungs, lymph nodse, tonsils, and thymus **(D)**, detected using qRT-PCR. Statistical significance is denoted by *p < 0.05.

After the HP-PRRSV challenge, piglets in all groups, except for those in groups 5 and 6, became viremic. The number of viremic piglets in group 4 was less than that in group 3 2–14 dpc (Figure 6A). Group 4 piglets had no viremic piglets detected 14 dpc, whereas group 3 piglets had until 21 dpc for viremia to disappear (Figure 6A). Compared with group 3 piglets, group 4 piglets displayed significantly lower serum viral load between 2 and 4 dpc and significantly lower viral load in the lungs and lymph nodes 21 dpc (Figures 6B,D). As shown in Figure 6C, the virus was detected in nasal swabs only from piglets in group 1 and group 2 for the duration of the challenge and peaked at 7 dpc, but there was no significant difference. Unexpectedly, no virus was detected in the thymus of group 4 and group 3 piglets.

### PNS Treatment Significantly Improves Immune Factor Expression in PRRSV-Inoculated PBMCs

**Figure 7:**
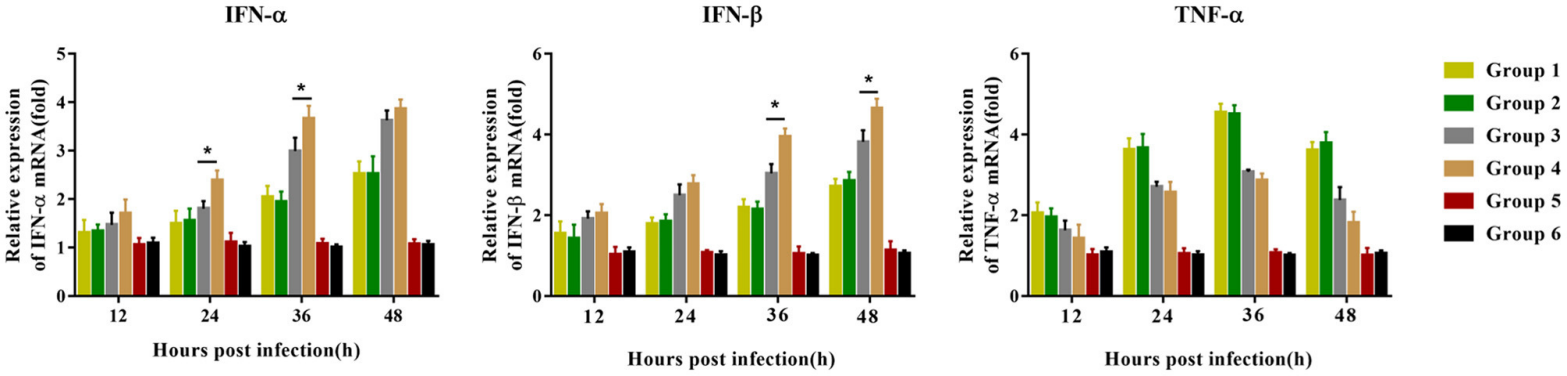
Immune-related gene expression in PBMCs re-stimulated with JXA1 (MOI of 0.05 TCID_50_ per cell) *in vitro*. PBMCs were collected from all piglets 28 dpv. Harvested PBMCs were incubated with JXA1 (MOI of 0.05 TCID_50_ per cell) for 1 h at 37°C and then cultured in a fresh medium. Cells were collected for the detection of mRNA expression of immune-related genes using qRT-PCR with specific primers at the indicated hours post-infection. Statistical significance is denoted by *p < 0.05.

Before the challenge, peripheral blood lymphocytes were isolated and inoculated with PRRSV to detect the mRNA expression of immune cytokines. Compared with group 3 piglets, group 4 piglets demonstrated significantly upregulated mRNA expression of IFN-α 24–36 hpi, and IFN-β 36–48 hpi, but no difference in TNF-α mRNA expression was observed. Group 1 and group 2 piglets had the same trend in the expression levels of the three cytokines, but there was no significant difference between the two groups (Figure 7). No significant difference was observed in the expression of the three cytokines in the piglets of groups 5 and 6 12–48 hpi.

## Discussion

The present study evaluated the anti-PRRSV activity of PNS and its potential in improving the immune efficacy and protection of the PRRSV MLV against the HP-PRRSV challenge.

Before the evaluation of immunomodulatory effects, we first evaluated the activity of PNS against PRRSV, which may interfere with the immunogenicity of PRRSV MLVs. As shown in Figures 1, 2, treatment of PRRSV-infected Marc-145 cells with PNS significantly reduced virus titers by inhibiting viral replication. Furthermore, PNS suppressed virus reproduction in the JXA1-R, NADC30-like, and GM2-like strain infected Marc-145 cells (Figure 3). A similar finding that saponins inhibit PRRSV replication has also been recently reported (10). Ginsenoside Rg1, an effective ingredient in PNS, can inhibit the activation of NF-κB signaling triggered by PRRSV infection (10). The additional mechanism of suppression of PRRSV replication by PNS, if any, remains to be further elucidated. It is worth mentioning that to determine the inhibition effect in HP-PRRSV-infected Marc-145 cells treated with PNS, we have to consider the anti-PRRSV effect of PNS in animals, although the concentration of PNS in piglets fed PNS may be lower than the concentration required to inhibit the replication of PRRSV *in vitro*. The concentration in the blood of piglets fed with PNS was not evaluated.

Compared with vaccinated piglets, vaccinated piglets treated with PNS possessed higher PRRSV-neutralizing antibodies (Figure 5). Neutralizing antibodies exert anti-viral effects by reducing the adsorption and internalization of viruses (22). After infection with PRRSV, the body first produces antibodies directed against the nucleocapsid protein N and the envelope protein M and then generates antibodies against the envelope glycoprotein GP5 (23). Many studies have shown that neutralizing antibodies are generated against GP2a, GP3, GP5, and M proteins, but not against the N-protein (24–28). Antibodies produced in the early stage of infection with PRRSV generally cannot neutralize the virus, and neutralizing antibodies can only be detected after at least 3 weeks (29). In addition, the levels of neutralizing antibodies are low and delayed, and cannot prevent viral infection, inhibit virus replication, or eliminate viremia (7, 30). A similar finding indicated that PNS can increase the levels of IgG and IgM in piglets infected with PRRSV (31). IgG and IgM are both essential immunoglobulins for humoral immunity. Saikosaponin a and saikosaponin d prevent viral infections by activating immunoglobulins through a neutralizing mechanism (32). Unfortunately, we found that neutralizing antibodies produced by piglets immunized with oral PNS could not protect against heterologous strains (data not shown). Meanwhile, oral administration of PNS did not have a beneficial effect on reducing viremia and improving growth performance, as well as immune response, compared to non-immunized piglets.

Vaccination with the PRRSV-2 MLV protected piglets from viremia after the challenge. Compared with group 1 piglets, group 3 piglets had a smaller number of viremic piglets and significantly fewer serum viral load 2–21 dpc (Figures 6A,B). Oral administration of PNS significantly increased the levels of PRRSV viremia and tissue viral load in vaccinated piglets. Group 4 had a smaller number of viremic piglets 2–21 dpc, as well as significantly less PRRSV viremia 2–4 dpc and tissue viral load 21 dpc than group 3 (Figures 6A,B,D). The serum anti-PRRSV antibody levels in groups 3 and 4 were not significantly different 0–28 dpv (Figure 4F), suggesting that PNS did not affect PRRSV-2 MLV immunogenicity. Viremia decreased significantly 2–4 dpc in group 4, which may not be attributed to the anti-PRRSV activity of PNS, but rather to the immune response induced by PNS.

Type I interferons include IFN-α and IFN-β, which play a very important role in the host's innate and specific immunity against viruses. PRRSV can inhibit the production of IFN-α and IFN-β, which is considered to be one of the mechanisms of PRRSV immune evasion (33, 34). The recovery of IFN-α secretion may contribute to the establishment of the TH1 cell immune response and inhibit PRRSV replication (35, 36). PRRSV inhibits the expression of IFN-β in Marc-145 cells by inhibiting the phosphorylation of IRF3 and IFN-β promoter activity (37). However, we found that oral PNS administration can significantly enhance IFN-α and IFN-β levels induced by the PRRSV MLV (Figure 7). PNS can also partially restore the number of red blood cells in piglets infected with PRRSV (32), and red blood cells have powerful immune regulatory capabilities (38).

In conclusion, oral PNS administration in combination with MLV vaccination significantly increased the level of neutralizing antibodies to homologous PRRSV strain and improved lung lesion scores and reduced severity of lung lesions after the challenge. Oral PNS administration significantly enhances the expression of IFN-α and IFN-β mRNA, which may contribute to reduced viremia after the PRRSV-2 challenge. Our results show that PNS has the potential as an oral immune modulator supplement, which can enhance the protective effect of the PRRSV-2 MLV against HP-PRRSV viremia.

## Data Availability Statement

The original contributions presented in the study are included in the article/supplementary material, further inquiries can be directed to the corresponding author/s.

## Ethics Statement

The animal study was reviewed and approved by Laboratory Animal Committee of South China Agricultural University.

## Author Contributions

HY and ZY designed the experiment. YCa, QW, and JP performed the experiment. HY, ZY, YCa, JM, YCh, CQ, and CJ performed the animal experiments. YCa, YS, and MC analyzed the data. HY wrote the manuscript. HW and GZ checked and finalized the manuscript. All authors contributed to the article and approved the submitted version.

## Funding

This work was supported by the National Natural Science Foundation of China (No. 31872489), China Agriculture Research System of MOF and MARA.

## Conflict of Interest

ZY is employed by Zhongmu Institutes of China Animal Husbandry Industry Co., Ltd., China. The remaining authors declare that the research was conducted in the absence of any commercial or financial relationships that could be construed as a potential conflict of interest.

## Publisher's Note

All claims expressed in this article are solely those of the authors and do not necessarily represent those of their affiliated organizations, or those of the publisher, the editors and the reviewers. Any product that may be evaluated in this article, or claim that may be made by its manufacturer, is not guaranteed or endorsed by the publisher.
